# Antibiotics Prescription by Spanish General Practitioners in Primary Dental Care

**DOI:** 10.3390/antibiotics10060703

**Published:** 2021-06-11

**Authors:** Laura Domínguez-Domínguez, Alfonso López-Marrufo-Medina, Daniel Cabanillas-Balsera, María Carmen Jiménez-Sánchez, Victoria Areal-Quecuty, José López-López, Juan. J. Segura-Egea, Jenifer Martin-González

**Affiliations:** 1 Endodontic Section, Department of Stomatology, University of Sevilla, C/Avicena s/n, 41009 Sevilla, Spain; alfonso.lopezmarrufo.m@gmail.com (A.L.-M.-M.); dcabanillas@us.es (D.C.-B.); jimenezsanchez6@gmail.com (M.C.J.-S.); victoria.areal@cabimer.es (V.A.-Q.); segurajj@us.es (J.J.S.-E.); 2 Department of Odontostomatology, Faculty of Medicine and Health Sciences (Dentistry), Barcelona University Dental Hospital, University of Barcelona, 08007 Barcelona, Spain; 18575jll@gmail.com

**Keywords:** antibiotic, antibiotics resistance, dentistry, general practitioner, endodontics, prescription habits, primary care

## Abstract

The aim of this study was to analyze the antibiotics prescription habits, both prophylactically and therapeutically, of Spanish general dental practitioners in the management of endodontic infections in primary care. Two hundred Spanish general dental practitioners were asked to respond to a survey on indications for antibiotics prescription in the treatment of endodontic infections, being 190 general dentists (95%) included in the study. Data were analyzed using descriptive statistics and the chi-square test. The average duration of antibiotics therapy was 6.5 ± 1.0 days. In patients without medical allergies, most of them (97%) selected amoxicillin as the antibiotic of the first choice, alone (51.1%) or associated with clavulanic acid (45.8%); in patients with penicillin allergies, the drug of choice was clindamycin 300 mg (70%). For cases of symptomatic irreversible pulpitis, 44% of the respondents prescribed antibiotics, in the scenario of prophylactic antibiotic prescription, up to 27% of the general dentists prescribe according to non-current guidelines (1 g 1 h before or 1 g 1 h before and 1 g 1 h after) in non-indicated cases (16% in patients taking oral bisphosphonates). It is necessary to improve the antibiotic prescription habits of Spanish general dentists in endodontics.

## 1. Introduction

One of the most serious threats to Spanish and global public health is the emergence of infections caused by bacterial strains resistant to treatment with antibiotics. A key factor in the emergence of bacterial resistance to antibiotics is the misuse of antimicrobial agents by healthcare workers. Approximately 10% of antibiotics dispensed in primary care are prescribed by dentists [1,2]. Currently, there is clear evidence that dentists in their daily clinical practice prescribe mainly painkillers and antibiotics, and their antibiotic prescribing habits are often unnecessary and inadequate [1,3]. Although these inadequate prescription patterns in dentists have been identified worldwide [4], it is worth noting that in Spain, they have been identified not only in dentists with clinical practice [5,6] but also in students in their final year of the Degree in Dentistry [3]. Inappropriate use of antibiotics is not only associated with increased antibiotic resistance but also with an increased risk of potentially fatal anaphylactic reactions, thus exposing people to unnecessary side effects [3]. Therefore, there is a lack of public and professional awareness of the risks of inappropriate antibiotic use, both in dentistry and in other aspects of health care [7].

Endodontic infections are very prevalent, being characterized by a polymicrobial microbiota, involving a combination of facultative Gram-positive, Gram-negative, and strictly anaerobic bacteria [1,8]. They are usually characterized by a rapid onset and a short duration of about 2 to 7 days if the cause is treated or eliminated [1]. The vast majority can be successfully treated by eliminating the source of the infection and establishing favorable conditions through local treatment, drainage, or dental extraction, with no need for oral or systemic antibiotics [9]. Recently, the European Society of Endodontics (ESE) [10] and the Spanish Endodontic Association (AEDE) [11] have developed a guide to establish when the administration of systemic antibiotics is indicated in conjunction with endodontic therapy. Thus, to justify the need for systemic antibiotics in endodontic infections, after adequate endodontic disinfection and drainage of abscesses in case of inflammation, it is necessary to have systemic involvement (fever > 38 °C, general malaise, lymphadenopathies, or trismus), progression of the infection (increased inflammation, cellulitis, and osteomyelitis), the persistence of the infection or patient medically compromised [2,12]. Therefore, adjuvant systemic antibiotics are unnecessary and therefore contraindicated in cases of symptomatic irreversible pulpitis, necrotic pulps, symptomatic apical periodontitis, acute apical abscesses without systemic involvement, or in patients with a normal immune system, as well as in cases of asymptomatic apical periodontitis [2,13].

Incorrect dosage and excessive duration of treatment are among the factors that have been associated with the development of bacterial resistance to antibiotics due to improper prescribing [8]. The prescribed dose of antibiotic should be such that it reaches the therapeutic values without causing harm to the host [3]. According to the ESE [10] and the AEDE [11], the recommended loading dose should be double the maintenance dose to reach a concentration 3–4 times higher than the minimum inhibitory concentration (MIC) from the start of treatment. The duration of antibiotic treatment will be determined by the clinical improvement of symptoms, and treatment should be stopped when there is evidence of improvement [10,11]. Beta-lactam antibiotics (penicillin V and amoxicillin) are the antibiotics of choice for endodontic infections, being amoxicillin, alone or in combination with clavulanic acid, the most prescribed antibiotic in Europe to treat infections of endodontic origin [1,4,6,14,15,16].

Several studies have analyzed through surveys the antibiotic prescription habits of Spanish endodontists [1] and oral surgeons [6]. However, no studies have analyzed the prescribing habits of primary care general dentists. For all of the above reasons, given the seriousness of the problem and the fact that antibiotic resistance is a serious and urgent global risk to public health, the aim of this study is to investigate the antibiotics prescription habits, both prophylactically and therapeutically, of Spanish general dental practitioners in the management of endodontic infections in primary care.

## 2. Materials and Methods

### 2.1. Study Population

In this cross-sectional descriptive survey, 200 Spanish general dental practitioners were asked to respond to a survey on indications for systemic antibiotics in the treatment of endodontic infections, as well as on antibiotic prophylaxis in endodontics. The only requirements for participation were to be a practicing dentist in primary care. The dentists who responded and completed the survey satisfactorily were 190 (95%), all of whom were included in the study. The dentists who participated in the survey did so anonymously, voluntarily, and without compensation.

### 2.2. Questionnaire

**Figure 1:**
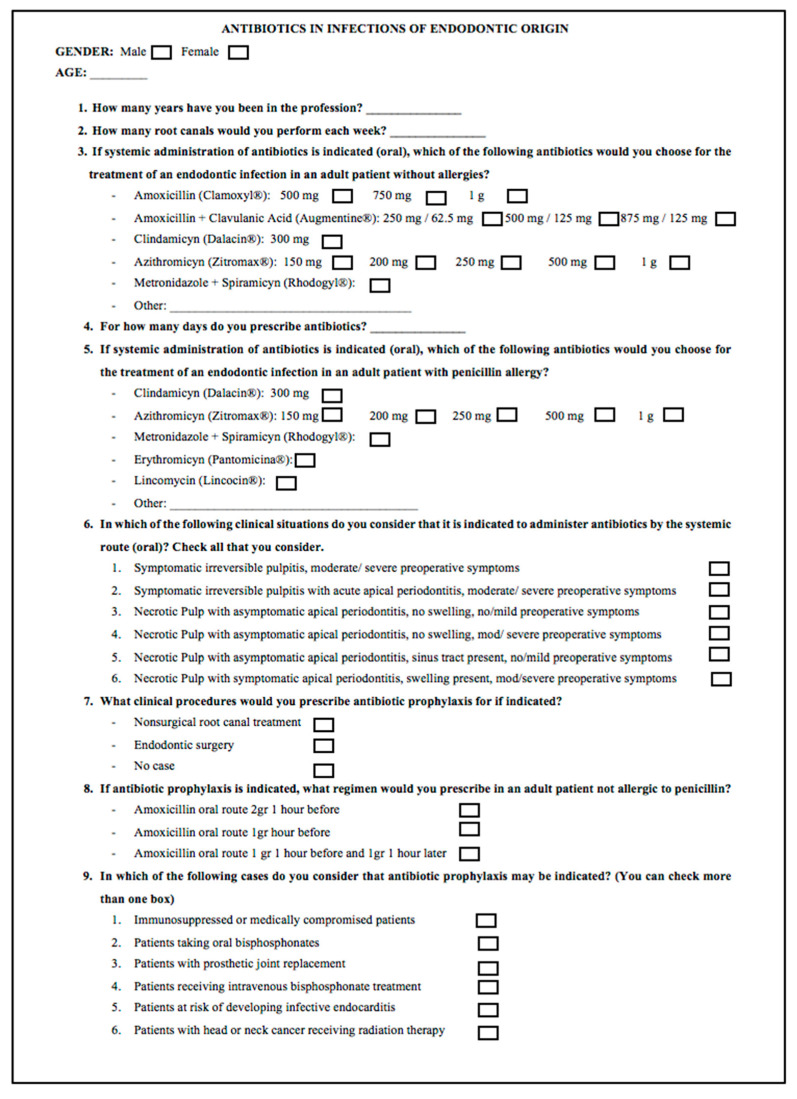
Survey on antibiotic prescribing habits in the treatment of endodontic infections.

The questionnaire (Figure 1) was based on those formulated in previous surveys in the United States [8,17] and Spain [1,3,5]. In addition, questions on prescribing habits in cases of antibiotic prophylaxis were added.

### 2.3. Data Collection and Statistical Analysis

For the collection of the data, the Excel program version 15.40 (Microsoft Corp., Redmond, WA, USA) was used, describing them by means of frequency tables. The numerical variables are expressed as mean ± standard deviation. The data were analyzed using descriptive statistics and the chi-square test. ANOVA and the Tukey test for independent samples were used to assess differences between groups. Significant differences were considered when *p* < 0.05.

## 3. Results

### 3.1. Participation and Description of Respondents

The demographics of the 190 participants are described in Table 1. The women surveyed (*n* = 124) represented 65.3% and the men 34.7% (*n* = 66). The average age of the respondents was 30.5 (SD = 7.8), with the age group ≤ 30 being the most predominant (65.8%). Referring to the average experience as a dentist was 74.0 ± 88.7 months, with low experience being more prevalent (65.8%). The number of weekly root canals was 4.3 (SD = 4.7).

### 3.2. Preferred Antibiotics

**Figure 2:**
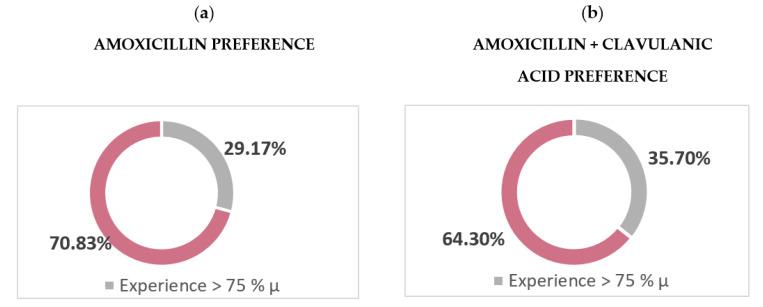
Percentages of antibiotic prescriptions according to experience as general dentists, where *p* > 0.05.

The majority of respondents (96.8%) selected amoxicillin as the first-choice antibiotic in patients without medical allergies, either alone (51.1%) or in combination with clavulanic acid (45.8%) (Table 2). Dentists with more experience prescribe amoxicillin associated with clavulanic acid as the first choice, while those with less experience prescribe it alone, with non-significant differences (*p* > 0.05) (Figure 2). Grouping all the data, the first-choice antibiotic for general dentists was amoxicillin 875 mg/clavulanic acid 125 mg (29.5%).

Regarding the antibiotic preferences of dentists in patients with penicillin allergies, clindamycin 300 mg was the first drug of choice (70%) (Table 3).

### 3.3. Duration of Antibiotic Treatment

**Figure 3:**
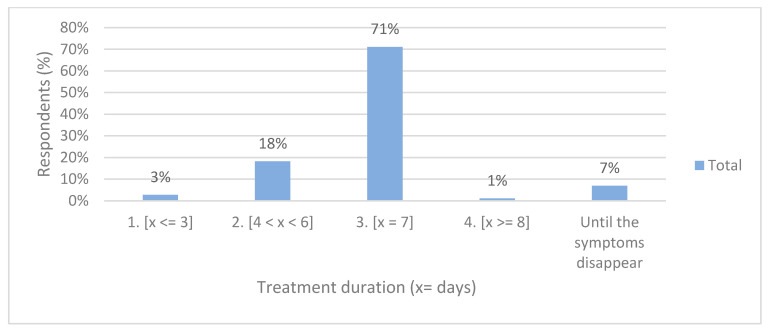
Distribution of responses by treatment duration.

The average duration of antibiotic therapy was 6.5 ± 1.1 days. Most respondents (71%) prescribed antibiotics for 7 days. No highly experienced general dentist eliminates antibiotic therapy when systemic symptoms cease. Only 7% of dentists, particularly all of them with less experience, prescribe antibiotics until systemic symptoms disappear (Figure 3). Differences according to experience in responses by the duration of treatment were not significant (*p* = 0.15).

### 3.4. Antibiotic Prescription in Each Clinical Situation

**Figure 4:**
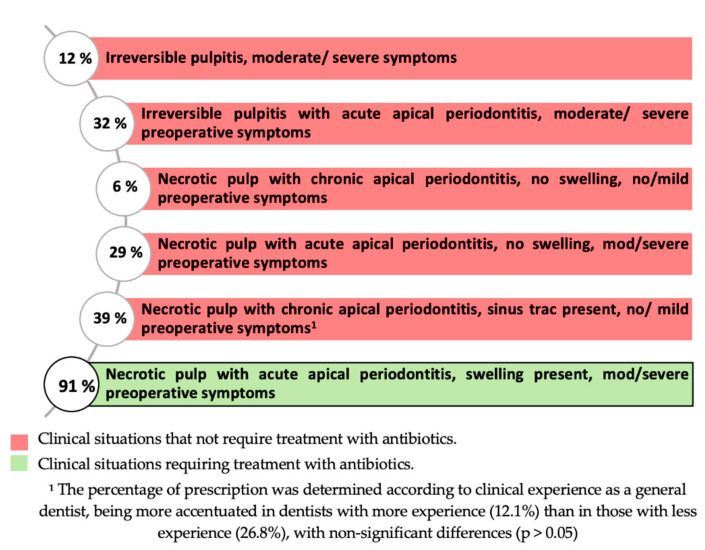
Antibiotic prescription according to the type of endodontic infection.

Figure 4 represents the percentage of participants prescribing antibiotics for various pulp-periapical diagnoses. More than 90% of dentists agree that antibiotics should be prescribed in cases of necrotic pulp with symptomatic apical periodontitis, apical abscess, and moderate/severe symptoms, while 39.0% prescribe antibiotics in cases of necrotic pulp with asymptomatic apical periodontitis, fistulous tract, and mild/symptomatic symptoms, with this prescription being more accentuated in more experienced dentists, with non-significant differences (*p* > 0.05).

### 3.5. Antibiotic Prophylaxis in Endodontics

The results obtained on clinical procedures, guidelines, and patients with indications for antibiotic prophylaxis are described in Table 4. More than three-quarters of general dental practitioners (80.5%) agree that antibiotic prophylaxis is necessary in case of periapical surgery. When comparing the antibiotic prophylaxis guidelines followed by general dentists according to the degree of experience, it can be observed that the percentage of misprescription was higher in those with lower experience (12.5%) with non-significant differences (*p* > 0.05). Dental practitioners prescribe antibiotic prophylaxis mainly for patients at risk of infectious endocarditis (97.4%). Misprescription of antibiotic prophylaxis in patients being treated with oral bisphosphonates was higher in the group with less experience. No significant differences were found (*p* > 0.05).

## 4. Discussion

This is the first study that reflects the current state of knowledge and prescribing habits of Spanish general dentists on the indications of systemic antibiotics in the treatment of pulp-periapical infections and antibiotic prophylaxis in the area of endodontics. The analysis of the data shows that a great majority of people select correctly the type of antibiotic for the treatment of these infections, as well as for the prophylaxis, but there is a high rate of generalists who prescribe antibiotics inaccurately.

The sample of the present study (*n* = 190) was similar to previous studies [1,3,5], with a high general response rate (95%). For a better analysis of the results, the sample was divided into two groups according to experience: a high experience group represented by 65 dentists (34.2%) and a low experience group represented by 125 dentists (65.8%). These results could not be compared with other studies because these parameters were not established in those studies.

Recently, the European Society of Endodontics (ESE) and the Spanish Endodontic Association (AEDE) [11] have developed a guide to establishing the indications of systemic antibiotics in conjunction with endodontic therapy, as well as a guideline for prophylaxis. These have been the reference used to interpret the results obtained [10,11].

The results of the present study coincide with the rest of Spanish surveys [1,3,5,6] and with those carried out in other European countries [4,14,16], in which the antibiotic of the first choice to treat infections of endodontic origin in patients without medical allergies is amoxicillin (96.8%), alone (51.1%) or in combination with clavulanic acid (45.8%). The European Society of Endodontics [10] and the Spanish Endodontic Association [11] recommend only amoxicillin as the first-choice antibiotic in non-allergic and non-immunocompromised patients, reserving its combination with clavulanic acid in cases where previous therapy has not been effective and in immunocompromised patients. Comparing the results of first-choice antibiotics according to experience, it was found that the more experience dentists have, the more inappropriate they prescribe because they associate amoxicillin with clavulanic acid as a first choice (*p* > 0.05). This could be explained by the lack of updating based on scientific evidence by more experienced dentists who continue to apply obsolete clinical guidelines.

In patients with allergies to β-lactams, the results of this study coincide with the rest of the Spanish surveys [1,3,5,6] and with the position of the European Society of Endodontics [10] and the Spanish Endodontic Association (AEDE) [11], in which the drug of the first choice is clindamycin 300 mg (70%). However, the antibiotic of choice for penicillin allergies varies throughout the world and even between regions within the same [4].

The average duration of antibiotic therapy was 6.5 ± 1.1 days, and most of the respondents (71%) prescribed antibiotics for 7 days, coinciding with the rest of the Spanish surveys [1,3,5,6]. No highly experienced dentist eliminates antibiotic therapy when systemic symptoms cease. This indicates that the misconception that bacterial infections require a “full course” of antibiotic therapy still exists, but there is no scientific evidence that a one-week period is necessary to treat endodontic infections. The guide for the duration of the treatment with antibiotics is the clinical improvement so that the antibiotic therapy will last until the symptoms have resolved, being essential to the follow-up of the patients. It is recommended to prescribe antibiotics for 3 days and review the patient after 3 days to determine whether treatment should be suspended or continued [10,11]. The percentages of erroneous prescriptions are very high, highlighting the lack of knowledge of current scientific evidence, which is higher with more experience (*p* = 0.15).

With regard to endodontic clinical situations requiring antibiotics as coadjutants in endodontic treatment, even though they are only indicated in the last situation proposed in the survey (necrotic pulp with acute apical periodontitis and systemic involvement), the present study shows high percentages of prescription in clinical situations that do not require it. The high percentage of prescriptions in the last clinical situation (91.1%) coincides with previous studies [1,3,5,6]. However, it should be noted that the percentage of dentists prescribing antibiotics in the fifth situation (38.9%), necrotic pulp with chronic apical periodontitis, no/moderate symptoms, and sinus tract present, is higher than reported in previous studies conducted with endodontists [1] and dentists in postgraduate training in endodontics [5]. In addition, this erroneous prescription was more pronounced in high experienced dentists (*p* > 0.05). Therefore, high experienced dentists prescribe more erratically than those with less experience.

Clinical recommendations on the use of prophylactic antibiotics in compromised patients have been changing [2], which may explain why it has not been studied in previous studies. Regarding endodontic procedures where antibiotic prophylaxis is indicated, 80.5% of general dentists agree that it is necessary in case of periapical surgery. The recommended prophylaxis regimen [10] in patients without medical allergies is 2 g of amoxicillin taken by mouth 1 h before the procedure. This was the preferred prophylaxis regimen in the present study (71.1%). However, the percentage of dentists prescribing an erroneous pattern is very high (26.8%), with a higher percentage of incorrect prescriptions in dentists with lower clinical experience (*p* > 0.05).

Antibiotic prophylaxis should be considered in immunosuppressed patients with a blood neutrophil count < 500/µL and, in patients with *locus minoris resistentiae*, it is very important to assess each case individually. The erroneous prescription of antibiotic prophylaxis in patients being treated with oral bisphosphonates was high (16.3%), being higher in the group with less experience (*p* > 0.05). Therefore, despite the fact that ignorance of the antibiotic prophylaxis regimen is high in both groups, it is more prevalent with less clinical experience.

The results of the present study show that it is necessary and essential to developing new strategies to improve the knowledge and prescribing habits of general dentists in Spain in the treatment of pulp-periapical conditions and the indication of prophylaxis in endodontic clinical settings. To this end, the teaching of educational programs on antibiotic prescription and patient management must be improved in official studies in dentistry and in continuing education programs offered by the various professional associations of dentists, as well as by the various scientific societies in the sector.

## 5. Conclusions

Most general dentists working in primary care are selecting the appropriate antibiotic and indicating it when it really is needed in the treatment of endodontic infections. However, there are still dentists who prescribe antibiotics inappropriately and do not follow the position of the European Society of Endodontics (ESE) and the Spanish Endodontic Association (AEDE). The use of antibiotics for minor infections, or in some cases in patients without infections, as well as unnecessarily prescribed antibiotic prophylaxis, could be a major contributing factor to the global problem of antimicrobial resistance. It is necessary to propose measures to improve the correct prescription of systemic antibiotherapy in the treatment of endodontic infections.

## Figures and Tables

**Table 1:** Description of participants.

**Sex**	***n* (%)**
Male	66 (34.7%)
Female	124 (65.3%)
**Age (y)**	**30.5 ± 7.8**
≤30	125 (65.8%)
30–40	33 (17.4%)
≥40	31 (16.3%)
**Experience as a dentist (months)**	**74.0 ± 88.7**
High experience (> 75% µ)	65 (34.2%)
Low experience (< 75% µ)	125 (65.8%)
**Number of weekly root canals**	**4.3 ± 4** **.7**

**Table 2:** Antibiotic preference in patients with no medical allergies.

Antibiotics	*n* (%)
**Amoxicillin**	
500 mg	48 (25.3%)
750 mg	30 (15.8%)
1000 mg	19 (10.0%)
**Amoxicillin/Clavulanic acid**	
225/62.5 mg	3 (1.6%)
500/125 mg	28 (14.7%)
875/125 mg	56 (29.5%)
**Clindamicym**	
300 mg	1 (0.5%)
**Azithromicyn**	0 (0%)
**Metronidazole/Spiramicyn**	
125 mg/750.000 UI	3 (1.6%)
**Other**	1 (0.5%)
**Blank answer**	1 (0.5%)

**Table 3:** Antibiotic preference in patients with medical allergies.

Antibiotics	*n* (%)
**Clindamicym**	
300 mg	133 (70.0%)
**Azithromicyn**	
150 mg	4 (2.1%)
200 mg	0 (0%)
250 mg	4 (2.1%)
500 mg	22 (11.6%)
1000 mg	0 (0%)
**Metronidazole/Spiramicyn**	14 (7.4%)
**Erythromicyn**	9 (4.7%)
**Lincomicyn**	0 (0%)
**Other**	1 (0.5%)
**Blank answer**	0 (0%)

**Table 4:** Clinical procedures, regimens, and patients with indications for antibiotic prophylaxis in endodontics.

**Clinical Procedures**	**N (%)**
Nonsurgical root canal treatment	51 (26.8%)
Endodontic surgery	153 (80.5%)
No case	34 (17.9%)
**Prophylaxis regimens in patients without medical allergies**	**N (%)**
Amoxicillin oral route 2 g 1 h before	135 (71.1%)
Amoxicillin oral route 1 g 1 h before ^1^	33 (17.4%)
Amoxicillin oral route 1 g 1 h before and 1 g 1 h later ^1^	18 (9.5%)
**Patients with indication for antibiotic prophylaxis**	**N (%)**
Immunosuppressed patients	133 (70.0%)
Patient taking oral bisphosphonates ^1^	31 (16.3%)
Patients with prosthetic joint replacement	84 (44.2%)
Patients receiving intravenous bisphosphonate treatment	49 (25.8%)
Patient at risk of developing infective endocarditis	185 (97.4%)
Patients with head or neck cancer receiving radiation therapy	61 (32.1%)

^1^ Percentage of antibiotic prescriptions according to experience as a general dentist have been calculated for these variables, where *p* > 0.05.
